# A joint latent class model for classifying severely hemorrhaging trauma patients

**DOI:** 10.1186/s13104-015-1563-4

**Published:** 2015-10-24

**Authors:** Mohammad H. Rahbar, Jing Ning, Sangbum Choi, Jin Piao, Chuan Hong, Hanwen Huang, Deborah J. del Junco, Erin E. Fox, Elaheh Rahbar, John B. Holcomb

**Affiliations:** Division of Clinical and Translational Sciences, Department of Internal Medicine, The University of Texas Medical School at Houston, The University of Texas Health Science Center at Houston, Fannin St, Houston, TX USA; Division of Epidemiology, Human Genetics and Environmental Sciences, School of Public Health, The University of Texas Health Sciences Center at Houston, Pressler St, Houston, TX USA; Department of Biostatistics, The University of Texas MD Anderson Cancer Center, Holcombe Blvd, Houston, TX USA; Division of Biostatistics, School of Public Health, The University of Texas Health Sciences Center at Houston, Pressler St, Houston, TX USA; Epidemiology and Biostatistics, College of Public Health, University of Georgia, Buck Road, Athens, GA 30602 USA; Division of Acute Care Surgery, Department of Surgery, Center for Translational Injury Research, The University of Texas Health Science Center at Houston, Fannin St, Houston, TX USA; Department of Biomedical Engineering, Wake Forest University, Winston-Salem, NC USA

**Keywords:** Induced censoring, Joint model, Latent variable, Massive transfusion, Mixture, Trauma

## Abstract

**Background:**

In trauma research, “massive transfusion” (MT), historically defined as receiving ≥10 units of red blood cells (RBCs) within 24 h of admission, has been routinely used as a “gold standard” for quantifying bleeding severity. Due to early in-hospital mortality, however, MT is subject to survivor bias and thus a poorly defined criterion to classify bleeding trauma patients.

**Methods:**

Using the data from a retrospective trauma transfusion study, we applied a latent-class (LC) mixture model to identify severely hemorrhaging (SH) patients. Based on the joint distribution of cumulative units of RBCs and binary survival outcome at 24 h of admission, we applied an expectation-maximization (EM) algorithm to obtain model parameters. Estimated posterior probabilities were used for patients’ classification and compared with the MT rule. To evaluate predictive performance of the LC-based classification, we examined the role of six clinical variables as predictors using two separate logistic regression models.

**Results:**

Out of 471 trauma patients, 211 (45 %) were MT, while our latent SH classifier identified only 127 (27 %) of patients as SH. The agreement between the two classification methods was 73 %. A non-ignorable portion of patients (17 out of 68, 25 %) who died within 24 h were not classified as MT but the SH group included 62 patients (91 %) who died during the same period. Our comparison of the predictive models based on MT and SH revealed significant differences between the coefficients of potential predictors of patients who may be in need of activation of the massive transfusion protocol.

**Conclusions:**

The traditional MT classification does not adequately reflect transfusion practices and outcomes during the trauma reception and initial resuscitation phase. Although we have demonstrated that joint latent class modeling could be used to correct for potential bias caused by misclassification of severely bleeding patients, improvement in this approach could be made in the presence of time to event data from prospective studies.

## Background

Hemorrhagic shock accounts for the largest proportion of mortality occurring within the first few hours of trauma center care, over 80 % of operating room deaths after major trauma and almost 50 % of deaths in the first 24 h of trauma treatment [1]. Due to rapidly changing multi-system responses to injury in a relatively short-term period, highly dynamic treatment regimes with blood transfusion are necessary and make comparative effectiveness research in this area very challenging. In blood transfusion medicine, however, there are no established or universally accepted measures to quantify blood loss or the severity of continuing hemorrhage. To compensate for the lack of quantitative metrics for bleeding severity, a single binary surrogate, namely massive transfusion (MT) stratification, became entrenched in the trauma literature, which is historically defined as the replacement of one’s total blood volume by transfusion of 10 or more units of red blood cells (RBCs) within 24 h of admission. This definition has been routinely used to investigate when to initiate a MT protocol, or as a stratification variable to account for potential confounding or effect modification when comparing the effectiveness of different resuscitation protocols [2–8]. However, there is a growing recognition of the pitfalls associated with the use of MT as a surrogate for bleeding severity and the need to replace this poor proxy [9–11]. The shortcomings associated with this classical definition are that it excludes patients who died of hemorrhage-related causes before (1) sufficient numbers of units of blood transfused (e.g., 10th of RBCs) within the specified post-admission time frame (e.g., 24 h) to achieve successful resuscitation, and (2) interventions to stop further blood loss (surgical repair of damaged blood vessels and tissue) could be completed.

Several groups have tried to develop better definitions for MT to ameliorate these shortcomings. A recent international forum highlighted twelve different definitions for MT; the most common being ≥5 or 6 RBCs within 4–6 h [12]. While the time period has been shortened from 24 h, this definition continues to exclude early deaths and does not account for the variability in additional blood products or other hemostatic interventions. An alternative approach has been considering the rate of transfusions. Savage et al. [13] defined “critical administration thresholds” (CAT) of ≥3 units of RBCs per hours to identify hemorrhaging patients. However, the CAT definition is still limited to RBC transfusions and does not account for plasma, platelet transfusions or crystalloids and colloids. More recently, Rahbar et al. [14] reported that 4 units of any resuscitative fluid including blood products, crystalloids and colloids, coined as the “resuscitation intensity”, within the first 30 min were predictive of 6 h mortality in their study. While these definitions are greatly improved from the classical definition of MT, the predictive analysis is still based on simple logistic regressions, which can be viewed as inadequate due to misclassification in the presence of death or informative dropouts [9]. In trauma care, these issues are critical because patient misclassification could result in increased risk of unnecessary blood transfusion or waste of limited and expensive blood resources.

In this article we propose a model-based classification approach for trauma patients. In the past decades, latent class (LC) modeling has been applied in various fields of sciences [15–19]. The goal of LC analysis is to take observed measures (e.g., presence of symptoms or markers of disease) and define a variable that is not directly observable the latent variable (e.g., disease status). These methods have been extended to jointly analyze longitudinal quantitative marker and survival outcome (or informative dropout process), which typically combine a mixed model for longitudinal data and a survival model depending on the latent class [20–24]. Rahbar et al. [11] were the first to apply a LC model to classify patients with severe hemorrhage. This class of models assumes that the dependency between the risk of event and the trajectory of the biomarker is entirely captured by a LC structure rather than by individual random effects. This can avoid many of the numerical complexities of the shared random-effects model under the conditional or so-called ‘local’ independence assumption. These methods are particularly useful for characterizing heterogeneous populations to more accurately guide clinical decision making.

**Fig. 1:**
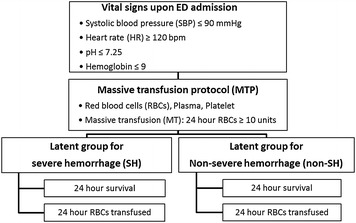
Diagrams for activation of massive blood transfusion protocol. Actual decision making for massive blood transfusion involves several vital signs at hospital admission and potential risks of blood transfusion and early mortality. The LC-based approach incorporates all these factors while MT is simply based on total utilization of RBCs

A unique challenge in analyzing trauma transfusion data is that a terminating or informative censoring event such as death prevents further intervention with blood transfusion. In our example, the total amount of RBC units transfused prior to death or within 24 h of admission is dependent upon the duration of a trauma patient’s hemodynamically unstable survival. Therefore, the observed blood amount during resuscitation is possibly correlated with patients’ survival. Such a dependency, also known as induced censoring, may produce spurious associations and misleading inference if not correctly addressed. To appropriately adjust for a similar induced dependency in medical cost analysis, Lin [25] proposed a linear regression model, accompanied by an inverse probability censoring weighting (IPCW) method. In this article, we consider a LC-based approach that utilizes comprehensive information on patient’s presentation, blood usage and survival outcome, with application to a retrospective trauma transfusion study [26]. Specifically, as an alternative to MT classification, using a logistic regression model we introduce a binary latent variable for severe hemorrhage (SH) that classifies severely injured trauma patients who may require massive blood transfusion. The class-specific logistic models for blood product utilization and survival status are then specified under the conditional independence (CI) assumption given each class membership. A benefit of the proposed approach is its ability to incorporate many observable quantities, such as vital signs upon emergency department (ED) admission, into all of these modeling components, as illustrated in Fig. (1), which may better reflect practical complexities and support establishment of a protocol for massive blood transfusion.

Therefore, our goal is to use a LC model to account for induced censoring and correct potential misclassification associated with MT. This research extends the previous work by Rahbar et al. [11] to develop an improved class of LC models that could be used to characterize SH patients. In addition, we will compare the predictive models developed by the new LC-based classification for SH with the traditional MT definition. The remainder of this paper is organized as follows. First, the retrospective trauma data are briefly described. The next section describes the statistical model for the biomarker and dropout processes. The performance of our method is evaluated using both simulated data and the data example. A concluding remark is provided in the last section.

### The retrospective trauma transfusion study

This work was motivated by data from a retrospective multi-center trauma transfusion study, which enrolled transfused trauma patients admitted to 16 level 1 trauma centers in the US between July 2005 and June 2006 [26]. Included in the study were 1574 adult trauma patients who arrived from the scene and received at least 1 unit of RBCs in the ED, irrespective of mechanism of injury. Patient characteristics, including age, sex and race, admission vital signs, such as systolic blood pressure (SBP), heart rate (HR), respiratory rate (RR), temperature, hemoglobin (Hgb), and international normalized ratio (INR), Glasgow Coma Scale (GCS), transfusions, admission clinical laboratory tests, prevalence of comorbidity, trips to the operating room and outcome data such as 6- and 24-h mortality and cause of death, were collected from each site and entered into a database at the Department of Epidemiology and Biostatistics, The University of Texas Health Science Center at San Antonio. Given that many patients were intubated upon arrival or in the ED, the respiratory rate was coded as 0 to account for the poor respiratory state. Units of RBCs, platelets, and plasma were adjusted to standard units and totaled at 6 and 24 h after admission. Crystalloid and colloid amounts were similarly recorded. Ventilator, ICU, and hospital-free days were calculated based on a stay of 30 days. Cause of death was categorized as multiple organ failure, truncal hemorrhage, head injury, airway problems, or others, and validated at each site.

For the analysis, among 1574 patients, 471 with full observations on SBP, HR, Hgb, and pH were included. Main characteristics (in total and MT vs. non-MT) were summarized in Table 1. The median age was 36 (first and third quartiles 25–52.5) years, and 350 patients (74.3 %) were male. Based on the conventional definition of MT, 211 (44.8 %) were MT and 260 (55.2 %) were non-MT. Some patient characteristics, such as base deficit, injury severity score and blood products usage, were substantially different across MT and non-MT. Out of all 471 patients, 68 (14.4 %) died in 24 h and 122 (25.9 %) died in 30 h. Among those who died within 24 and 30 h, there were 17 (25.0 %) and 47 (38.5 %) non-MT patients, respectively

**Table 1:** Summary characteristics of trauma patients in the retrospective study

Patient characteristics	Total	MT	Non-MT
	(*n* = 471)	(*n* = 211)	(*n* = 260)
	Mean (SD)^a^	Mean (SD)^a^	Mean (SD)^a^
Mortality			
Mortality, 0–24 h	68 (14.4 %)	51 (24.2 %)	17 (6.5 %)
Mortality, 0–30 h	122 (25.9 %)	75 (35.5 %)	47 (18.1 %)
Clinical outcomes			
Ventilation days	5.57 (9.99)	5.79 (9.39)	5.44 (10.34)
Intensive care unit days	8.55 (11.75)	9.98 (12.19)	7.38 (11.28)
Hospital days	17.42 (20.98)	20.68 (24.23)	15.09 (18.03)
Patient characteristics			
Age (year)	41.04 (19.17)	40.48 (18.62)	41.50 (19.62)
Gender (male)	350 (74.3 %)	163 (77.2 %)	187 (71.9 %)
Penetrating injury	37.24 (29.39)	42.82 (33.27)	32.27 (24.46)
Systolic blood pressure (mmHg)	112.25 (35.446)	103.46 (32.74)	119.38 (36.01)
Diastolic blood pressure (mmHg)	70.72 (24.11)	69.76 (22.78)	71.61 (25.29)
Heart rate (bpm)	106.26 (27.46)	115.41 (27.40)	98.83 (25.23)
Respiratory rate	21.42 (7.28)	22.31 (8.41)	20.78 (6.29)
Temperature (°C)	35.91 (1.05)	35.85 (1.26)	35.96 (0.86)
pH	7.25 (0.14)	7.21 (0.16)	7.28 (0.11)
International normalized ratio	1.44 (0.76)	1.56 (0.75)	1.33 (0.76)
Base deficit	−8.23 (6.11)	−10.05 (6.50)	−6.78 (5.37)
Glasgow Coma Scale	10.53 (6.70)	9.30 (5.52)	11.54 (7.38)
Injury severity score	27.68 (15.27)	32.35 (16.03)	23.87 (13.50)
Blood products usage			
RBC, 0–6 h (units)	8.95 (10.92)	16.91 (12.11)	2.49 (1.99)
RBC, 0–24 h (units)	11.67 (12.32)	21.41 (12.71)	3.77 (2.17)
Plasma, 0–6 h (units)	4.86 (6.86)	9.51 (7.75)	1.08 (2.16)
Plasma, 0–24 h (units)	6.87 (8.90)	13.09 (9.76)	1.83 (3.06)
Platelets, 0–6 h (units)	2.57 (6.04)	5.45 (8.01)	0.23 (1.43)
Platelets, 0–24 h (units)	4.54 (9.43)	9.44 (12.26)	0.56 (2.01)
Plasma/RBC ratio, 0–24 h (units)	0.51 (0.59)	0.63 (0.37)	0.41 (0.71)
Platelet/RBC ratio, 0–24 h (units)	0.24 (0.40)	0.43 (0.40)	0.09 (0.32)

^a^Mean (SD) is for continuous variables. For categorical (or binary) variables, count (%) are reported as indicated by the % sign

## Methods

### Model and notation

We assume that there are two latent homogeneous subgroups and label this latent variable as SH versus non-SH, where SH patients are more likely to require activation of a MT protocol. From a statistical perspective, the methodology can be easily generalized to problems with more than two latent classes. Suppose that we have a random sample of *n* patients. For patient $i\in \{1,\ldots ,n\}$, let $g_i=(g_{i1},g_{i2})$, where $g_{ik}$ is an indicator of membership of class $k=1,2$, and suppose that we observe the biomarker readings $y_i$ and the survival indicator $w_i$ at 24 h of hospital admission. By conditional independence, it is assumed that $y_i$ and $w_i$ are independent given the membership $g_i$. The baseline covariates or treatment information will be incorporated into $v_i$ for the membership model or $x_i$ for the class-specific models. Denoting the conditional distribution of *A* given *B* as [*A*|*B*] and the entire set of parameters by $\Psi$, the log-likelihood can be decomposed as

$$\begin{aligned} l(\Psi ) =\sum _{i=1}^n \log \left( \sum _{k=1}^2 [g_{ik}=1| v_i] [y_i|x_i,g_{ik}=1] [w_i|x_i, g_{ik}=1] \right) . \end{aligned}$$

The proposed model can be further described as follows. The probability $\pi _{i1}=1-\pi _{i2}$ that subject *i* belongs to class 1 can be modeled as a function of a vector of covariates $v_i$ in a logistic regression with

1 $$\begin{aligned} \pi _{i1}(v_i)={\textit{P}}(g_{i1}=1|v_i)=\frac{\exp (v_i^T\alpha )}{ 1+ \exp (v_i^T\alpha )}, \end{aligned}$$

where $\alpha$ is the vector of regression parameters. Next, we assume that the probability of death for class $k=1,2,$ depends on the covariates $x_i$ through a binary logistic regression:

2 $$\begin{aligned} {\textit{P}}(w_i=1|g_{ik}=1,x_i)=\frac{\exp (x_i^T\gamma _k)}{1+\exp (x_i^T\gamma _k)}, \end{aligned}$$

where $\gamma _k$ is the *k*th class-specific coefficient for $k=1,2$. Here, $w_i=1$ corresponds to death within 24 h, 0 otherwise. Finally, suppose the response variable $y_i$ depends on $x_i$ through a linear model: given $g_{ik}=1$,

3 $$\begin{aligned} y_i=x_i^T \beta _k+\epsilon _{ik},\quad \epsilon _{ik}\sim N(0,\sigma ^2), \end{aligned}$$

where $\beta _k$ is a vector of regression coefficients in class *k*. We assume equal variance for each component in order to avoid the unboundedness of the mixture likelihood. In our trauma data, $y_i$ represents the logarithm of cumulative amount of RBCs consumed up to 24 h or time of death, whichever occurs first, and $w_i$ is the survivorship status at 24 h of hospital admission. However, the exact amount of RBCs transfused at 24 h is observable only when a patient survives at least for 24 h (i.e., $w_i=0$), otherwise, it is censored at the time of death or dropout. Such a phenomenon is common with medical cost data, in which some study subjects are not followed for the full duration of interest so their total costs are unknown for the subjects who are censored. To correct the associated selection bias, Lin [25] adapted an inverse probability of censoring weighted (IPCW) technique to a linear model. This method, however, is not applicable to our situation, because full assessment to survival outcomes is limited with the retrospective data. Instead, we assume that

4 $$\begin{aligned} \exp (y_i^\textsc {obs})\sim \text {Uniform}[0,\exp (y_i)], \quad \text {if} \quad w_i=1, \end{aligned}$$

that is, the observed amount of RBCs transfused $(y^\textsc {obs}_i)$ is uniformly distributed with true amount $y_i$ as the upper boundary. Through a simulation study, we examine the effect of a biased estimation in which censored observations are not adjusted with (4). Although (4) is an untestable assumption, we demonstrated that it is helpful in reducing potential bias caused by induced censoring.

### Parameter estimation

Estimation of the unknown parameters in the proposed mixture model can be performed using a maximum likelihood method. Based on the observed data $\mathbf {O}=\{(y^\textsc {obs}_i,w_i,v_i,x_i);i=1,\ldots ,n\}$, the observed likelihood function for $\Psi =\{(\alpha ,\beta _k,\gamma _k,\sigma );k=1,2\}$ is

5 $$\begin{aligned} &L (\Psi )\\ &\quad=\prod _{i=1}^n [y_i,w_i|x_i,v_i,\Psi ] \nonumber\\ &\quad=\prod _{i=1}^n \left( \sum _{k=1}^2 \pi _{ik}(v_i) [y_i|g_{ik}=1,x_i,\Psi ] [w_i|g_{ik}=1,x_i,\Psi ] \right)\nonumber\\ &\quad=\prod _{i=1}^n \Bigg ( \sum _{k=1}^2 \frac{\exp (g_{ik} v_i^T\alpha )}{ 1+ \exp (v_i^T\alpha )} \left[ \left\{ \phi \left(\frac{y^\textsc {obs}_i-x_i^T\beta _k}{\sigma }\right)\right.\right.\\ &\qquad\qquad\qquad\qquad \times\left. \left( \frac{1}{1+\exp (x_i^T\gamma _k)} \right) \right\}^{I(w_i=0)} \nonumber \\&\quad\qquad \times \left.\left.\bigg \{ \int _{y_i^\textsc {obs}}^\infty e^{-(u-y_i^\textsc {obs})} \phi \bigg (\frac{u-x_i^T\beta _k}{\sigma}\bigg )du \bigg ( \frac{\exp (x_i^T\gamma _k)}{1+\exp (x_i^T\gamma_k)} \bigg ) \bigg \}^{I(w_i=1)} \right]\right),\end{aligned}$$

where $\phi (\cdot )$ is a standard normal density. The third equality in (5) follows from conditional independence assumption between $y_i$ and $w_i$ given all covariates and the latent variable.

However, it would be cumbersome to maximize the observed-data log-likelihood (5) analytically due to complexities by the presence of mixing parameters and the non-linearity caused by censored observations. To simplify the estimation procedure, we introduce a random variable $z_i$ for unobservable $y_i$ for the drop out of patient *i* by death status. We treat latent variables $g_i$ and $z_i$ as missing data and invoke the expectation-maximization (EM) algorithm to maximize the log-likelihood. Given $g_i$ and $z_i$, the complete-data log-likelihood is

6 $$\begin{aligned} l_c(\Psi )&=\sum _{i=1}^n\sum _{k=1}^2 (v_i^T\alpha ) g_{ik} -\sum _{i=1}^n \log \left\{ 1+ \exp (v_i^T\alpha )\right\} \\\nonumber \\&\quad -\frac{n}{2} \log \sigma ^2 \\ \nonumber \\&\quad -\frac{1}{2\sigma ^2}\sum _{i=1}^n\sum _{k=1}^2 g_{ik}(1-w_i)(y^\textsc {obs}_i-x_i^T\beta _k)^2 \\\nonumber &\quad-\frac{1}{2\sigma ^2}\sum _{i=1}^n\sum _{k=1}^2 g_{ik}w_i(z_i-x_i^T\beta _k)^2 +\sum _{i=1}^n\sum _{k=1}^2 g_{ik}[(x_i^T\gamma _k)w_i\\ \nonumber &\quad-\log\{1+\exp (x_i^T\gamma _k)\}]. \end{aligned}$$

In EM algorithm, we alternate between expectation step (E-step) and maximization step (M-step). In the E-step of the $(s+1)$th iteration, we evaluate the expectation of the complete-data log-likelihood (6), conditional on the observed data $\mathbf {O}$ and the current parameter estimate, say $\Psi ^{(s)}$. This is equivalent to calculating the expected values of all the functions of $g_i$ and $z_i$ that appear in the complete-data log-likelihood. Let $\tilde{E}(\cdot )$ represent such an expectation and $\tilde{g}_{ik}=\tilde{E}[g_{ik}|\Psi ]$. The posterior class-membership probability is then

7 $$\begin{aligned} \tilde{g}_{ik}&=\frac{[g_{ik}=1|v_i][y^\textsc {obs}_i,w_i|x_i,v_i,g_{ik}=1]}{[y^\textsc {obs}_i,w_i|x_i,v_i]} \\ \nonumber &=\frac{\pi _{ik}(v_i)[y^\textsc {obs}_i|x_i,g_{ik}=1][w_i|x_i,g_{ik}=1]}{\sum _{k=1}^2 \pi _{ik}(v_i)[y^\textsc {obs}_i|x_i,g_{ik}=1][w_i|x_i,g_{ik}=1]}. \end{aligned}$$

Based on the assumption (4), the $z_i$’s have the following class-specific distribution:

8 $$\begin{aligned} &p(z_i|y^\textsc {obs}_i,g_{ik}=1,\Psi ) \\ &\quad =\frac{p_k(z_i,y^\textsc {obs}_i|\Psi )}{\int \nolimits _{y^\textsc {obs}_i}^\infty p_k(u,y^\textsc {obs}_i|\Psi )du} \\ \nonumber &\quad =\frac{e^{-(z_i-y^\textsc {obs}_i)} \phi \left( \frac{z_i-x_i^T\beta _k}{\sigma }\right) }{ \int _{y^\textsc {obs}_i}^\infty e^{-(u-y^\textsc {obs}_i)} \phi \left( \frac{u-x_i^T\beta _k}{\sigma }\right) du}, \end{aligned}$$

for which we calculate $\tilde{E}_k[z_i^r|\Psi ]= \int _{y^\textsc {obs}_i}^\infty z_i^r p(z_i|y^\textsc {obs}_i,g_{ik}=1,\Psi )dz_i$ for $r=1,2$ and $k=1,2$. Let $\mathcal {Q}(\Psi ;\Psi ^{(s)}) = \tilde{E}_{g,z}[ l_c(\Psi )|\Psi ^{(s)}]$ be the expected complete-data log-likelihood at the *s*th step, given by

$$\begin{aligned} \mathcal {Q}(\Psi ;\Psi ^{(s)})&=\sum _{i=1}^n\sum _{k=1}^2 (v_i^T\alpha ) \tilde{g}_{ik}^{(s)} \\ \nonumber &\quad -\sum_{i=1}^n \log \left\{ 1+ \exp (v_i^T\alpha )\right\} - \frac{n}{2} \log \sigma ^2 \\&\quad -\frac{1}{2\sigma ^2}\sum _{i=1}^n\sum _{k=1}^2 \tilde{g}_{ik}^{(s)} \{ (1-w_i) ( y^\textsc {obs}_i-x_i^T\beta _k)^2 \\ \nonumber &\quad +w_i \tilde{E}_k[ ( z_i-x_i^T\beta _k)^2 |\Psi ^{(s)})] \} \\&\quad +\sum _{i=1}^n\sum _{k=1}^2 \tilde{g}_{ik}^{(s)} [(x_i^T\gamma _k)w_i \\ \nonumber &\quad -\log \{1+\exp (x_i^T\gamma _k)\}], \end{aligned}$$

which is maximized in the M-step with respect to $\Psi$; that is, $\Psi ^{(s+1)}={\arg \max }_{\Psi } \mathcal {Q}(\Psi ;\Psi ^{(s)})$.

In our normal-mixture model, updating model parameter $\Psi$ in the $(s+1)$th step is tantamount to calculating

$$\begin{aligned} \alpha ^{(s+1)}&= \text {arg max}_{\alpha } \sum _{i=1}^n \left[ (v_i^T\alpha ) \tilde{g}_{ik}^{(s+1)}\right. \\&\quad\left.- \log \left\{ 1+ \exp (v_i^T\alpha )\right\} \right] , \\ \beta ^{(s+1)}_k&=(X^T W_k^{(s+1)}X)^{-1}X^TW_k^{(s+1)}\tilde{y}_k^{(s)} ,\\ \gamma ^{(s+1)}_k&=\text {arg max}_{\gamma _k}\sum _{i=1}^n[ \tilde{g}_{ik}^{(s+1)} (x_i^T\gamma _k)\\&\quad w_i-\tilde{g}_{ik}^{(s+1)} \log \{1+\exp (x_i^T\gamma _k)\}],\\ \sigma ^{2(s+1)}&=\frac{1}{n}\sum _{i=1}^n\sum _{k=1}^2 \tilde{g}_{ik}^{(s+1)} \{(1-w_i)(y_i^\textsc {obs}-x_i^T\beta _k^{(s+1)})^2 \\&\quad+w_i \tilde{E}_k[ ( z_i-x_i^T\beta _k^{(s+1)})^2 |\Psi ^{(s)}] \}, \end{aligned}$$

where $X=(x_1,\ldots ,x_n)^T$, $W_k^{(s+1)}$ is an $n\times n$ diagonal matrix with diagonal elements $\{\tilde{g}_{ik}^{(s+1)},i=1,\ldots ,n\}$, $\tilde{y}_k^{(s)}=(\tilde{y}_{1k}^{(s)},\ldots ,\tilde{y}_{nk}^{(s)})^T$, where $\tilde{y}_{ik}^{(s)}=y^\textsc {obs}_{i}$ if $w_i=0$, otherwise, $\tilde{y}_{ik}^{(s)}=\tilde{E}_k[z_i|\Psi ^{(s)}]$. The EM-based maximum-likelihood algorithm updates $\beta _k$ by a weighted least squares estimate in the M-step as $\phi (\cdot )$ is a normal density. The EM algorithm is initiated from an initial value $\Psi ^{(0)}$, after which one oscillates between the E-step and M-step until convergence is achieved. In order to avoid local maxima for the examples in this paper, the maximization process was repeated 20 times with random starting values. Thus, the reported estimates represent the maximizer over the 20 maximizations. The use of multiple starting points is quite standard in application of LC models and not terribly onerous for practical purpose. For the examples in this paper, the algorithm converged fairly quickly, and, for the most part, the global maximum was not hard to find.

### Standard error estimation

We estimate standard errors of the estimated class-conditional model and the mixing parameters, using the empirical observed information matrix under the EM algorithm framework,

9 $$\begin{aligned} I_c(\hat{\Psi };\mathbf {O})=\sum _{i=1}^n S_c(\mathbf {O}_i;\hat{\Psi })S_c(\mathbf {O}_i;\hat{\Psi })^T, \end{aligned}$$

where $S_c(\mathbf {O}_i;\hat{\Psi })$ represents the *i*th individual complete-data score function with respect to the vector of parameters $\Psi$, evaluated at the maximum likelihood estimate $\hat{\Psi }$. The covariance matrix of the parameter estimates is then approximated by the inverse of the empirical Fisher information (9). The appeal of this approach is that all the terms in (9) are by-products of the M-step and provide a reasonable way to estimate standard errors for all model parameters. Wald’s test can then be performed based on the estimated variance-covariance matrix.

### Classification

Once the model is fitted, patients can be classified into one of several latent subgroups. In our data example, latent groups can have substantive meaning, such as a group of SH patients for future MT protocol. Although we focus on a two-mixture model, the proposed methodologies can be easily generalized to problems with $K\ge 2$ latent classes. Patients’ membership in various subgroups will be determined based on estimated posterior probabilities. We have that ${\textit{P}}(g_{ik} = 1) = \pi _{ik}$, termed prior probability; this class probabilities $\pi _{ik}$ represent the likelihood that *i*th patient belongs to group *k* but without using information from characteristics of patients, blood usage and survival status. In contrast, the posterior probability of patient *i* belonging to the *k*th group is given by (7). This represents how likely is that the *i*th patient belongs to group *k*, taking into account the observed response $y^\textsc {obs}_i$ as well as the survival status $w_i$ of that patient. Using these posterior probabilities, we classify patient *i* into class *k* if and only if $\tilde{g}_{ik}=\max _j\{\tilde{g}_{ij}\}$. However, in situations where two or more posterior probabilities are almost equal, classification becomes nearly random, which could result in misclassifications. In general, we can vary the number of latent groups *K* and explore the sensitivity of the classification to the number of latent classes considered. Also, we may use several cut-off points for posterior probabilities and examine whether the results remain consistent.

## Results

### Numerical study

In order to assess performance of LC analysis for identifying subpopulations we conducted a simulation study, in which 1000 data sets were simulated, each containing measurements and covariate information from 250 and 500 patients. Mimicking the retrospective trauma study, the LC variable in the model is assumed to split the patient into two latent subgroups. Component probabilities for the LC mixture model follow the logistic model:

$$\begin{aligned} \pi _{i1}(v_i)=1-\pi _{i2}(v_i)= \frac{\exp (\alpha _0+\alpha _1 v_i)}{1+\exp (\alpha _0+\alpha _1 v_i)}, \end{aligned}$$

which involves one covariate $v_i\sim N(0,1)$. We let $\alpha =(\alpha _0,\alpha _1)^T=(0.5,1)^T$ so that approximately 60 % of patients belong to class 1. For the binary survival status, the logistic regression is based on a binary random variable $x_i \sim \text {Bernoulli(0.5)}$:

$$\begin{aligned} {\textit{P}}(w_i=1|g_{ik}=1,x_i)=\frac{\exp (\gamma _{0}^{(k)}+\gamma _{1}^{(k)}x_i)}{1+\exp (\gamma _{0}^{(k)}+\gamma _{1}^{(k)}x_i)}, \quad k=1,2. \end{aligned}$$

The parameters in these models, $\gamma ^{(k)}=(\gamma _0^{(k)},\gamma _1^{(k)})^T$, differ for both latent classes with $\gamma ^{(1)}=(1,-1)^T$ and $\gamma ^{(2)}=(-1,1)^T$, corresponding to mortality rates of 62 and 38 % for class 1 and class 2, respectively. Finally, logarithm of observed RBCs at 24 h were generated from the class-specific linear model that allowed censoring: when $g_{ik}=1$,

10 $$\begin{aligned} y_i^\textsc {obs} = \beta _0^{(k)}+\beta _1^{(k)}v_i+\beta _2^{(k)} x_i+\Delta _i^{(k)}+\epsilon _i, \quad \epsilon _i\sim N(0,\sigma ^2), \end{aligned}$$

where

$$\begin{aligned} \exp (\Delta _i^{(k)})=\left\{ \begin{array}{ll} 1, &{} \text { if } w_i=0, \\ \text {Uniform}[0,1],&{}\text { if } w_i=1. \end{array}\right. \end{aligned}$$

That is, true cumulative RBC units can be measured only when the patient is alive ($w_i=0$), otherwise, observed values will be lower than or equal to the true measurement but at random. We let $\beta ^{(1)}=(\beta _0^{(1)},\beta _1^{(1)},\beta _2^{(1)})^T=(\log (15),-1,1)^T$ and $\beta ^{(2)}=(\beta _0^{(2)},\beta _1^{(2)},\beta _2^{(2)})^T=(\log (8),1,-1)^T$, so that patients in class 2 will receive generally smaller amount of cumulative RBC units. We consider three scenarios with $\sigma =0.5,$ 1 and 2, respectively. In this setting, class 1 may represent the SH subgroup which requires more blood products transfusion. By contrast, conventional MT definition will identify MT patients by the rule: $\exp (y^\textsc {obs}_i)\ge 10$.

Table 2 contains the results of our simulation study. We calculated the bias of estimates, the empirical standard error (SSE), the average of estimated standard errors (ASE). Besides comparing the mean estimates and true values of the parameters through the bias, we also reported the mean squared error (MSE) that simultaneously involves bias and precision. Simulation results show that bias seems negligible and SEEs and ASEs match reasonably well for all model parameters in three scenarios. Both bias and standard error become smaller as the sample size grows. For the estimation of $\sigma$, we observed some discrepancy between sample and estimated standard errors, but there is no significant impact on the estimation of other regression parameters of interest.

**Table 2:** Summary statistics for the simulation studies for the two-component LC mixture model under different scenarios ($\sigma =0.5,1,$ and 2) and two sample sizes (*n* = 250 and 500)

Scenario	Parameter	True	*n* = 250				*n* = 500			
			Est	SSE	ASE	MSE	Est	SSE	ASE	MSE
$\sigma =0.5$	$\alpha _0$	0.5	0.514	0.201	0.201	0.040	0.502	0.139	0.133	0.017
	$\alpha _1$	1.0	1.027	0.232	0.226	0.052	1.009	0.157	0.155	0.024
	$\beta _0^{(1)}$	2.708	2.708	0.071	0.073	0.005	2.710	0.050	0.050	0.002
	$\beta _1^{(1)}$	−1.0	−1.002	0.055	0.054	0.003	−1.002	0.038	0.037	0.001
	$\beta _2^{(1)}$	1.0	0.998	0.101	0.105	0.011	0.995	0.071	0.071	0.005
	$\beta _0^{(2)}$	1.609	1.605	0.141	0.133	0.017	1.610	0.096	0.094	0.009
	$\beta _1^{(2)}$	1.0	1.001	0.094	0.090	0.008	1.001	0.062	0.062	0.004
	$\beta _2^{(2)}$	−1.0	−1.001	0.156	0.151	0.022	−0.999	0.108	0.108	0.012
	$\gamma _0^{(1)}$	1.0	1.011	0.325	0.322	0.104	1.008	0.225	0.215	0.046
	$\gamma _1^{(1)}$	−1.0	−1.005	0.405	0.398	0.158	−1.012	0.281	0.270	0.073
	$\gamma _0^{(2)}$	−1.0	−1.015	0.389	0.388	0.150	−1.024	0.268	0.261	0.068
	$\gamma _1^{(2)}$	1.0	1.010	0.500	0.487	0.237	1.034	0.345	0.330	0.110
	$\log (\sigma )$	−0.693	−0.718	0.032	0.063	0.005	−0.703	0.022	0.044	0.002
$\sigma =1$	$\alpha _0$	0.5	0.522	0.272	0.267	0.072	0.507	0.187	0.185	0.034
	$\alpha _1$	1.0	1.034	0.281	0.283	0.079	1.009	0.190	0.183	0.034
	$\beta _0^{(1)}$	2.708	2.709	0.149	0.153	0.023	2.706	0.104	0.104	0.011
	$\beta _1^{(1)}$	−1.0	−1.006	0.112	0.113	0.012	−0.998	0.077	0.077	0.006
	$\beta _2^{(1)}$	1.0	1.001	0.206	0.203	0.043	1.003	0.147	0.145	0.021
	$\beta _0^{(2)}$	1.609	1.577	0.276	0.277	0.078	1.601	0.188	0.194	0.037
	$\beta _1^{(2)}$	1.0	0.986	0.185	0.182	0.033	1.002	0.124	0.121	0.014
	$\beta _2^{(2)}$	−1.0	−0.980	0.298	0.301	0.091	−0.995	0.206	0.213	0.045
	$\gamma _0^{(1)}$	1.0	1.005	0.366	0.348	0.121	1.005	0.253	0.251	0.063
	$\gamma _1^{(1)}$	−1.0	−1.003	0.443	0.415	0.172	−1.003	0.307	0.298	0.089
	$\gamma _0^{(2)}$	−1.0	−1.014	0.44	0.427	0.182	−1.012	0.308	0.310	0.096
	$\gamma _1^{(2)}$	1.0	1.016	0.558	0.516	0.266	1.021	0.385	0.400	0.160
	$\log (\sigma )$	0.0	−0.027	0.065	0.065	0.005	−0.012	0.045	0.042	0.002
$\sigma =2$	$\alpha _0$	0.5	0.568	0.505	0.538	0.294	0.524	0.372	0.388	0.151
	$\alpha _1$	1.0	1.108	0.406	0.431	0.198	1.046	0.278	0.289	0.085
	$\beta _0^{(1)}$	2.708	2.749	0.336	0.350	0.124	2.717	0.239	0.240	0.057
	$\beta _1^{(1)}$	−1.0	−1.051	0.262	0.266	0.073	−1.023	0.188	0.193	0.037
	$\beta _2^{(1)}$	1.0	1.020	0.436	0.450	0.203	0.996	0.311	0.322	0.104
	$\beta _0^{(2)}$	1.609	1.520	0.599	0.698	0.496	1.576	0.409	0.438	0.192
	$\beta _1^{(2)}$	1.0	0.951	0.406	0.453	0.207	0.971	0.271	0.284	0.081
	$\beta _2^{(2)}$	−1.0	−1.036	0.596	0.615	0.380	−1.008	0.418	0.422	0.178
	$\gamma _0^{(1)}$	1.0	1.001	0.483	0.458	0.209	1.017	0.345	0.313	0.098
	$\gamma _1^{(1)}$	−1.0	−1.014	0.564	0.532	0.284	−1.023	0.397	0.359	0.129
	$\gamma _0^{(2)}$	−1.0	−0.955	0.565	0.533	0.286	−1.002	0.404	0.395	0.156
	$\gamma _1^{(2)}$	1.0	0.962	0.686	0.652	0.427	1.004	0.484	0.476	0.227
	$\log (\sigma )$	0.693	0.652	0.130	0.067	0.006	0.674	0.092	0.047	0.003

*SSE* sample standard errors, *ASE* average of estimated standard errors, *MSE* mean squared error

As true value of $\sigma$ increases, the associated error term in model (10) has large variation and thus two latent subgroups are less separable. This was reflected in the increased magnitude of MSE with large $\sigma$. We also note that the proportions that true latent variable coincides with the MT class were about 66, 53 and 38 % for $\sigma =0.5$, 1, 2, respectively, when $n=500$. On the other hand, the corresponding proportions that the estimated posterior probability from (7) correctly predicts the latent class were about 82, 74, and 62 %, implying that the LC-based classification consistently outperforms naïve MT classification.

### Application to the data from the retrospective trauma transfusion study

We illustrate application of the proposed method to the data from the retrospective trauma study [26]. The proposed LC model was applied to identify severely hemorrhaging (SH) patients who might need intensive massive transfusion care, assuming that the trauma patients could be split into two or more latent subgroups. The baseline covariates used in our analysis include the following binary patients’ characteristics at admission: (1) systolic blood pressure (SBP) <90 mmHg; (2) heart rate (HR) ≥120 bpm; (3) pH <7.25 and (4) Hemoglobin (Hgb) <9. These covariates were selected by exploratory analysis and included in models (1)–(3), respectively. In addition, the 24-h blood products ratio, (5) plasma:RBC ratio and (6) platelet:RBC ratio, were considered as treatment information in models (2) and (3). These two variables are categorized as (ratio = 0), $(0<\text {ratio}\le 1)$, and $(\text {ratio}>1)$. From the observed data, twe can only observe the total amount of RBCs transfused at 24 h or up to death, whichever comes first.

The proposed LC model was also fitted for different numbers of classes. The values of BIC as the number of classes varied from 1 to 5 were 1689.6, 1362.7, 1366.8, 1368.4, and 1402.1 respectively, and the associated numbers of parameters were 25, 47, 73, 99, and 125. The one-class model is inferior compared with those with more latent classes. The two-class model has the smallest BIC value and may be the favored approach to the data. Hence, the analysis below was based on a two-mixture model for SH (class 1) versus non-SH (class 2). The class-membership probability, given SBP, HR, pH and Hgb, can be calculated through estimated coefficients of the logistic model (1). To predict the log-transformed 24-h cumulative RBC transfusion, we used a class-specific linear model (3) and treated 24-h survivorship as a binary response in class-specific logistic models (2), both based on the cumulative 24-h ratios (plasma:RBC and platelet:RBC ratios). The results of the joint LC analysis with (1)–(3) are summarized in Table 3. For comparison purposes, we also carried out separate analyses of the three component models with conventional MT definition.

**Table 3:** The maximum likelihood estimates (standard errors in parenthesis) of the LC mixture analysis

Variable	Estimates (SE)	
*Model 1: linear regression model for latent-class structure*		
Intercept	−0.791 (0.613)	
SBP <90	0.570 (0.618)	
HR ≥120	0.554 (0.621)	
pH <7.25	0.384 (0.590)	
Hgb <9.0	0.574 (0.760)	

	SH	Non-SH
*Model 2: class-specific linear models for RBC transfusion*		
Intercept	2.400 (0.427)	0.767 (0.138)
SBP <90	0.081 (0.261)	0.188 (0.101)
HR ≥120	−0.169 (0.294)	0.089 (0.102)
pH <7.25	0.216 (0.307)	0.132 (0.087)
Hgb <9.0	−0.046 (0.331)	0.105 (0.161)
Plasma:RBC (0 vs. (0,1])	0.682 (0.405)	0.942 (0.104)
Plasma:RBC (0 vs. >1)	−0.145 (0.516)	0.616 (0.111)
Platelet:RBC (0 vs. (0,1])	0.673 (0.313)	0.820 (0.095)
Platelet:RBC (0 vs. >1)	0.125 (0.404)	0.547 (0.174)

	SH	Non-SH
*Model 3: class-specific logistic models for traumatic death*		
Intercept	−0.797 (1.070)	−1.425 (1.279)
SBP <90	0.169 (0.817)	−1.732 (2.530)
HR ≥120	−0.384 (0.739)	0.451 (1.347)
pH <7.25	1.083 (0.830)	2.027 (1.185)
Hgb <9.0	0.079 (0.938)	−1.850 (3.283)
Plasma:RBC (0 vs. (0,1])	0.727 (1.244)	−1.131 (1.628)
Plasma:RBC (0 vs. >1)	−0.819 (1.468)	−1.837 (2.029)
Platelet:RBC (0 vs. (0,1])	−0.424 (1.056)	−0.044 (1.706)
Platelet:RBC (0 vs. >1)	−1.288 (1.627)	1.969 (1.874)

Overall, the SH group is characterized by significantly higher units of RBC transfusion than those of the non-SH group (nearly 3 times higher in logarithmic scale), representing that on average the SH patients received more than 10 units of RBCs within 24 h. The effects of the plasma:RBC ratio and the platelet:RBC ratio on the cumulative 24-h RBC transfusion and the dropout pattern show a clear difference by latent classification. In the SH subgroup, the higher ratios of plasma/RBC and platelet/RBC were consumed, the lower dropout (death) rates were obtained. The SH classification will depend on the magnitude of cut-off for posterior probability (7). Because the LC mixture model considered here only contains two latent groups, we merely need to look at one of the posterior probabilities, e.g., the posterior probability that the patient belongs to class 1. Based on this, the patients can be classified following the suggested cut-off values in Table 4. If the posterior probability lies between 0.45 and 0.55, it is uncertain to which group the patient can be classified. Only 9 out of 471 patients in the trauma data are in this situation. For the most patients, 450 (95.5 %), it is more clear into which group they can be classified as their posterior probability is above 0.60.

**Table 4:** Classification of patients based on the posterior probabilities

Posterior probability	Classification	No. of patients
0.80–1.00	Group SH	83
0.60–0.80	Likely group SH	34
0.55–0.60	Doubtful, maybe group SH	6
0.45–0.55	Uncertain	9
0.40–0.45	Doubtful, maybe group non-SH	7
0.20–0.40	Likely group non-SH	61
0.00–0.20	Group non-SH	271

When the SH and MT classifications are applied to the same patients, the observed data can be summarized in Table 5. By regarding SH as “true” binary bleeding status, sensitivity and specificity are 82.7 and 69.2 %, implying the possibility that a non-ignorable proportion of trauma patients unnecessarily received MT intervention. Among 68 patients who died before 24 h, a non-ignorable portion of patients (17, 25 %) were not classified as MT but the SH group included 62 patients (91 %) who died during the same period. Among 22 patients who were non-MT but classified as SH, 14 died before 24 h post admission, while only 3 out of 106 MT but non-SH patients died. Almost half of SH patients were characterized by early mortality and may be misclassified by the MT definition.

**Table 5:** Observed number of patients classified by the LC analysis and conventional MT classification

LC analysis	Conventional		Total
	Non-MT	MT	
Non-SH	238	106	344
SH	22	105	127
Total	260	211	471

**Fig. 2:**
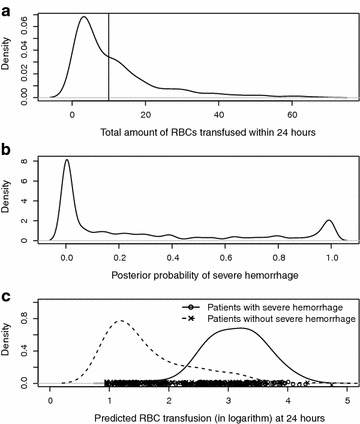
Distribution of **a** cumulative amount of RBCs, **b** posterior distribution of SH, and **c** predicted RBC units

Table 6 presents a summary of comparison between the MT patients who were in the SH and the non-SH groups. This shows that patients in SH and MT are characterized by higher death rates (46 %) and higher average RBC units transfused (22 units) and relatively lower average blood pressure (96 mmHg) at admission. In contrast, non-SH and MT patients had much lower death rate (3 %) and consumed fewer blood products than the SH group. Further comparisons are illustrated in Fig. 2. Patient identification by the observed amount of RBC appears to be less distinct, compared to classification by the posterior probability. Figure 2 further displays the distribution of the predicted RBC units given latent class, by replacing censored observations with their expectations under assumption (4). Clearly, patients in SH had higher RBC transfusions, ranging from 2 to 4, while RBC units in the non-SH group ranged from 1 to 4. This also indicates that patients who received a large volume of RBCs may not necessarily belong to the SH group.

**Table 6:** Summary statistics of 106 non-SH and 105 SH patients both in the MT group

	Non-SH and MT	SH and MT	P value
	Mean (SD)	Mean (SD)	
Patient characteristics			
Age (year)	40.01 (17.58)	40.94 (19.69)	0.720
Gender (male)	80 (76 %)	83 (78 %)	0.538
SBP (mmHg)	110.84 (30.79)	96.00 (33.09)	0.000*
pH	7.24 (0.14)	7.16 (0.17)	0.001*
Heart rate (bpm)	116.28 (27.17)	114.53 (27.72)	0.644
Respiratory rate	21.38 (7.83)	23.40 (8.97)	0.128
Hemoglobin	10.76 (2.68)	10.80 (2.68)	0.915
Death, 0–24 h	3 (3 %)	48 (46 %)	0.000*
Death, 0–30 h	19 (18 %)	56 (53 %)	0.000*
Blood product usage			
RBC, 0–6 h (units)	12.08 (7.39)	21.79 (13.90)	0.000*
RBC, 0–24 h (units)	16.18 (8.28)	26.67 (14.18)	0.000*
Plasma, 0–6 h (units)	7.66 (5.92)	11.38 (8.87)	0.001*
Plasma, 0–24 h (units)	11.20 (6.80)	14.98 (11.76)	0.005*
Platelets, 0–6 h (units)	5.15 (7.73)	5.75 (8.29)	0.586
Platelets, 0–24 h (units)	9.21 (10.10)	9.65 (14.14)	0.795
Plasma/RBC ratio, 0–24 h	0.71 (0.35)	0.55 (0.36)	0.001*
Platelet/RBC ratio, 0–24 h	0.48 (0.34)	0.37 (0.44)	0.037*

P values were obtained by comparing two subgroups

In practice, it is critical to expeditiously identify patients mostly likely to need activation of massive transfusion early in trauma care. Since clinician have been using MT definition as a way to identify early predictors of the need for MT protocol, one could use the new SH classification for identifying early predictors of SH. It is important to note that for both definitions, MT and SH, one needs to observe patients until hour 24-h. To demonstrate whether prediction models based on MT and SH differ, we performed a multivariable logistic regression using 325 patients and utilizing information from the following variables: SBP of less than 90 mmHg, Hgb of less than 11 g/dL, HR of greater than or equal to 120 bpm, temperature of less than 35.5 °C, INR of less than 1.5, and base deficit (BD) of less than 6. The Wald scores (Table 7) demonstrate the relative weighted influence of each variable, where INR, hemoglobin and heart rate appear to have significant predictability on SH. The predictive equation was $\log [p/(1-p)]=-0.5224+(0.3010\times \text {SBP})+(0.6628\times \text {HR}) +(0.9256\times \text {Hgb})+(1.6726\times \text {INR}) +(0.1057\times \text {Temperature})-(0.1648\times \text {BD})$ with a receivers operating characteristics (ROC) value of 0.73. The corresponding sensitivity, specificity, positive and negative predictive values are 69, 86, 38, and 96 %, respectively. We also reported the results from naïve analysis, where comparison was made between MT patients and non-MT patients. With respect to percentage of correct decision making, a positive INR (72 %) seems the best individual MT predictor followed by HR (69 %), SBP (68 %), Hgb (63 %). Importantly, all the individual rules remained significant negative predictors (NPV ≥75 %) with SH. Given the clinical utility of the laboratory parameters, particular work may be undertaken to obtain and validate these parameters within the LC framework as we proposed in this paper.

**Table 7:** Predictive models for SH and MT using a multivariate logistic regression

	SH				MT			
	Est.	SE	Wald	p value	Est.	SE	Wald	p value
(Intercept)	−0.5224	0.4348	−1.20	0.2296	0.0280	0.3910	0.07	0.9429
SBP <90	0.3010	0.3422	0.88	0.3790	0.3286	0.2895	1.14	0.2564
HR ≥120	0.6628	0.3195	2.07	0.0381	0.8856	0.2729	3.25	0.0012
Hgb <9.0	0.9256	0.4316	2.14	0.0320	0.9077	0.4611	1.97	0.0490
INR ≥1.5	1.6726	0.3301	5.07	0.0000	1.1429	0.3171	3.60	0.0003
Temp <35.5	0.1057	0.3597	0.29	0.7689	0.2092	0.2809	0.74	0.4564
BD <6	−0.1648	0.3697	−0.45	0.6558	0.0126	0.2904	0.04	0.9653

## Discussion

In this study we have used a joint latent class model to improve identification of severely hemorrhaging trauma patients. Because severely bleeding patients may benefit from rapid massive blood transfusion while those with mild blood loss could be potentially harmed by massive blood transfusion, their distinction is critically important but suffers from lack of predictive measurements. Our approach toward this end is to utilize posterior probabilities obtained by the LC method, given information from patient’s characteristics and survival information at 24-h post ED admission. The work presented here is considered as an extension of our earlier findings on this topic [11]. The advantage of the proposed method is that it uses admission vital signs to determine the latent variable representing the unknown amount of blood lost (i.e. degree of hemorrhage) in each submodel. Our model-based definition steers away from potential selection biases that could arise when a MT definition depends on a fixed quantity or rate of blood transfusion within a fixed time period. In this study, we found that out of a total of 68 patients who died before 24 h, 62 (91 %) were identified as SH. The fact that the MT classification misses about 66 % (=91–25 %) of these patients highlighted a major limitation of the classical definition. As a result, the MT definition is not a reasonable surrogate for building predictive models to guide massive blood transfusion protocol.

A number of trauma studies have examined other MT definitions, for example, ≥10 units in 6 h [2], ≥5 units in 4 h [7], or assigning patients who died of hemorrhage before receiving 10 units of RBCs into MT as well [27]. Alternatively there have been a few other approaches using rates of transfusions like CAT and ‘resuscitation intensity’ [13, 14]. However, all of these ad-hoc definitions could under- or over-represent patients who die early, and conversely, may include patients who do not present with critical hemorrhage but develop a need for MT intervention later during the course of their surgical and intensive care phase. Furthermore, it turns out that different MT definitions imply differences in transfusion practices [7, 8, 27]. It should be noted that selection bias from early mortality can be adjusted by using the IPCW technique [25], but such inclusion criteria, solely based on the amount of RBCs, may not fully reflect transfusion practice, which is involved with many other clinical factors, such as usage of other blood products.

Using our new SH definition, we have developed predictive models to identify early predictors of the need for MT protocol. Although this definition of SH could be further improved by using time to event data from prospective studies, the purpose of our effort in building predictive models using the definition of SH is to demonstrate differences in the coefficients of predictive models based on SH and MT definitions when using the same variables in these predictive models. The data presented in this paper clearly demonstrate a significant difference in the parameter estimates of these predictive models based on the SH and MT classifications.

It should be noted that this study is limited in being a retrospective review of data on trauma patients entered prospectively, and thus complete information, such as time to death, detailed timing of treatments and blood product utilization was partially available. Consequently, our approach has to rely on a relatively simple parametric model. With full time to event information (e.g., exact time of death), the mortality model in our proposal may be replaced by survival models, such as Cox model. Upon availability of such information, we can also relax the strict ‘local’ independence assumption, which is likely to be violated in practice. This approach may be applied to a more comprehensive data set from the PRospective Observational Multicenter Major Trauma Transfusion (PROMMTT) study, which is the first large scale, prospective study of trauma patients admitted directly from the injury scene to 10 level-1 trauma centers [10, 28]. The LC analysis with application to PROMMTT is currently undertaken by our research team, in which we will study broad endpoints of mortality, competing risks and adverse events, such as multisystem organ failure and acute lung injury, etc.

## Conclusions

An accepted definition of MT for trauma resuscitation is vital as it is commonly used to select a study population and drives trauma resuscitation guidelines. The classical MT definition of receiving ≥10 units of RBCs in 24 h of admission does not adequately reflect transfusion practice and outcome during the ED admission and initial resuscitation phase. Consideration of LC models permits useful joint analysis of biomarker and dropout data and enables bias-corrected estimation of the impact of prognostic features on the main endpoint associated with MT. It also permits full and exact posterior inference for predictive quantity of interest.

## Abbreviations

BD: base deficit
CAT: critical administration thresholds
CI: conditional independence
ED: emergency department
EM: expectation-maximization
GCS: glasgow coma scale
Hgb: hemoglobin
HR: heart rate
INR: international normalized ratio
IPCW: inverse probability of censoring weighted
LC: latent class
MT: massive transfusion
MTP: massive transfusion protocol
PROMMTT: PRospective Observational Multicenter Major Trauma Transfusion
RBC: red blood cells
RR: respiratory rate
SBP: systolic blood pressure
SH: severe hemorrhage
